# The impact of MRI slice thickness on the detection of spinal syndesmophytes in axial spondyloarthritis

**DOI:** 10.1186/s13075-025-03665-x

**Published:** 2025-11-14

**Authors:** Kalliopi Klavdianou, Daniel Benjamin Abrar, Alexander Dieter Mewes, Styliani Tsiami, Philipp Sewerin, Xenofon Baraliakos

**Affiliations:** 1 Rheumazentrum Ruhrgebiet Herne, Ruhr-University Bochum, Claudiusstr. 45, 44649 Herne, Germany; 2 Department of Rheumatology, “Asklepieion” General Hospital, Voula, Athens, 16673 Greece; 3 Medical Faculty, Department of Diagnostic and Interventional Radiology, University Dusseldorf, D-40225 Dusseldorf, Germany; 4 Radiology Munich, Burgstraße 7, 80331 Munich, Germany

**Keywords:** Syndesmophytes, MRI slice thickness, Radiography, Axial spondyloarthritis (axSpA)

## Abstract

**Background:**

Radiography is commonly used in clinical practice for detecting syndesmophytes in radiographic axial Spondyloarthritis (r-axSpA), while the ability of magnetic resonance imaging (MRI) to detect such bony structures is questionable due to its slicing technique. We aimed to assess the ability and performance for detection of syndesmophytes on MRI using different slice thicknesses and compare them with radiography in r-axSpA.

**Methods:**

MRI (T1-weighted (T1W) sequences) with slice thicknesses of 1–6 mm of the lower thoracic and lumbar spine were prospectively performed in patients with available radiographs. Each vertebral corner (VC) (anterior and posterior) from thoracic (Th11) to lumbar (L5) was assessed for presence/absence of syndesmophytes and/or fat lesions (FL, MRI only) by two experienced readers in independent MRI and radiography sessions and agreement was then reached in consensus.

**Results:**

A total of 1.204 VCs were assessed from 43 r-axSpA patients. Syndesmophytes were recorded in 19.3% VCs on radiography and in 38.3%, 37.5%, 34.8%, 33.7%, 31.4%, 28.7% VCs on MRI slice thicknesses of 1–6 mm, respectively (all *p* ≤ 0.001 vs. radiography). Although more syndesmophytes were recorded on MRI than radiography, MRI also missed 21%-31.3% syndesmophytes detected in radiography. Agreement with radiography was found in 72.6%, 73.8%, 75.9%, 76%, 77.3% and 78.5% on MRI slice thicknesses of 1–6 mm, respectively. FL were detected in 38.2%-39.2% in slice thicknesses 1–6 mm. Occurrence of FL was associated with better agreement between MRI and radiography findings.

**Conclusion:**

The thinner the MRI slices, the more syndesmophytes were detected compared to radiography, but the best agreement with radiography was found in the thicker slices. The presence of fat lesions on MRI was associated with better agreement with radiography for syndesmophyte detection.

**Supplementary Information:**

The online version contains supplementary material available at 10.1186/s13075-025-03665-x.

## Introduction

Axial spondyloarthritis (axSpA) is a chronic inflammatory rheumatic disease affecting the axial skeleton and comprises of two stages, radiographic (r-) and non-radiographic (nr-axSpA) [1]. AxSpA is characterized by inflammatory, osteodestructive and osteoproliferative changes in the sacroiliac joints (SIJs) and spine [2]. Syndesmophytes and ankylosis of the vertebral column are relevant structural changes indicating new bone formation in r-axSpA [3]. Syndesmophytes are thin bony outgrowths from the spinal ligaments attaching to adjacent vertebral bodies. Such bone proliferation starts at the vertebral rim and grows in the direction of the anulus fibrosus, parallel to the vertebral axis [3].

The modified Stoke Ankylosing Spondylitis Spine Score (mSASSS) [4], the most frequently used radiologic scoring method for r-axSpA [5], is heavily weighted by syndesmophytes and is used in clinical studies for assessment of radiographic progression in relation to treatment.

Inflammatory activity as depicted by magnetic resonance imaging (MRI) is predictive to structural changes seen subsequently on radiographs [6]. However, the exact sequence of this process is incompletely understood. The main hypothesis is that resolving inflammation associates with fat deposition [7–9]. Such fat lesions (FL), can be depicted by the T1-weighted (T1W) MRI sequences in vertebral bodies but also in the SIJs when present, and represent the strongest predictor for radiographic progression in axSpA. However, not all syndesmophytes develop at sites with preexisting fat or inflammation [7–10].

Radiography is the standard and most frequently employed technique for assessment of syndesmophytes in r-axSpA. While the lower part of the thoracic spine has been reported to be the most sensitive to change for assessment of syndesmophyte formation over time [11], the evaluation of the spine by radiography is mainly reported by inclusion of the cervical and lumbar spine only, due to the overlying soft tissues in the thoracic spine. Computed tomography (CT) has superior sensitivity and specificity compared to radiography, however, it is partly associated with higher radiation exposure, while both methods fail to detect bone marrow edema (BME) as a sign of inflammatory activity. In contrast, MRI is the best way to assess BME but the possibility for MRI to depict chronic changes such as syndesmophytes has not been yet determined.

This study aimed to assess the ability and performance of detection of syndesmophytes using different slice thicknesses on MRI and compare them with syndesmophytes depicted on radiography.

## Methods

Lower spine MRI and radiography were performed in patients with an established diagnosis of r-axSpA upon clinical indication for imaging due to low back pain, independent of treatment. The examinations were performed in a 3 Tesla MRI (Siemens Magnetom Prisma) system under the same protocol. MRI measurements consisted of a single sagittal T2 and a single sagittal T1W sequence. The T1W sequence was acquired with a slice thickness of 1 mm. Then, subsequent image series with increasing slice thicknesses, were reconstructed via the Multiplanar Reconstruction (MPR) tool of our inbound PACS software (IDS7, Sectra). Details of the MRI sequence parameters are summarized in Supplementary Table 1. Radiographs were available from the same patients in a sagittal position based on the protocol used in daily routine practice. Each VC (anterior superior and inferior and posterior superior and inferior) from the low thoracic (Th11) to the lumbar (L5) vertebral body was assessed for the presence/absence of syndesmophytes and presence/absence of FL in all MRI slice thicknesses and in radiographs (syndesmophytes only) by two experienced readers (one radiologist and one rheumatologist), independently. MRIs and radiographs were assessed in different reading campaigns, this means without the knowledge of the results of other imaging technique, respectively. Disagreements in findings for syndesmophytes and FL were finally solved in consensus and the findings with agreement were taken into account for the final analysis.

### Clinical information

The collected clinical information was age, sex, Bath Ankylosing Spondylitis Functional Index (BASFI) [12], disease duration and treatment at the timepoint of imaging.

### Statistical analysis

Descriptive measures (mean, median) are shown with SD, minimum and maximum or interquartile range (IQR). Frequencies and percentages are provided for categorical data (n). Comparison of medians of sums of affected lesions between different slice thicknesses and for each slice thickness versus radiography was performed using Wilcoxon signed rank test. Missing values, such as not evaluable VCs due to previous surgeries or missing clinical information, were not imputed. Results are always presented based on the available data for each parameter. All statistical tests were two-sided and a *p* < 0.05 was considered statistically significant.

## Results

### Patient characteristics

A total of 43 r-axSpA patients (81.4% males, mean age of 50 ± 15.4 years, median disease duration of 7.3 (IQR:15.5) years) were included. Table 1 shows the relevant clinical characteristics of the patients. Overall, a total of 1.204 VCs was assessed from all patients in both radiography and MRI. The VCs were assessed in all MRI slice thicknesses (1–6 mm).

**Table 1:** Clinical and demographic characteristics of the participants

Patient characteristics	All (*n* = 43)
males *n*, (%)	35 (81.4)
Age at MRI, years, mean ± SD	50 ± 15.4
median (IQR)	51.8 (13.1)
Disease duration at MRI, years, median (IQR)	7.3 (15.5)
BASFI, median (IQR)	6 (2.9)
Current Treatment *n* (%)	
bDMARD TNFi IL17i NSAIDs	35 (81.4) 30 (69.8) 5 (11.6) 8 (18.6)

bDMARD, biologic Disease-modifying antirheumatic drug; IL17i, interleukin 17 inhibitor; MRI, Magnetic resonance imaging; NSAIDs, Non-steroidal anti-inflammatory drugs; TNFi, Tumour necrosis factor (TNF)-alpha inhibitor

### Agreement in imaging evaluation between readers

Any discrepancies between readers were seen in 977 (9.5%) of the scored VCs. In detail, discrepancies were seen in 874 (9.7%) of the assessed VCs in the MRI and in 103 (8.6%) of assessed VCs in radiography. Most discrepant cases on MRI syndesmophytes were found in thinner slices (*n* = 180 discrepant findings in slice thickness of 1 mm).

### Overall comparison of syndermophytes on MRI and radiography

**Fig. 1:**
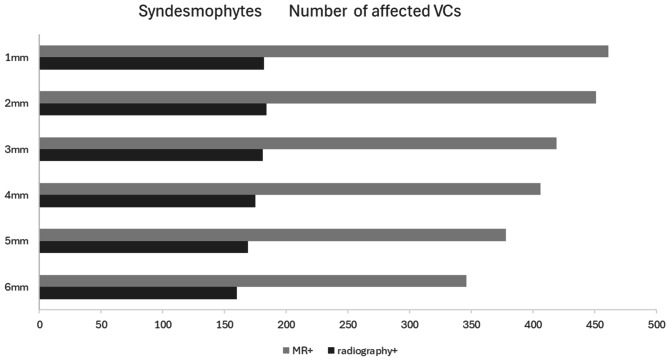
Distribution of VCs affected with syndesmophytes in different slice thicknesses of MRI. Radiography bar charts show the number of MRI detected syndesmophytes also seen in radiography MRI: Magnetic Resonance Imaging; VC: Vertebral Corner

Syndesmophytes in both the thoracic and lumbar spine were detected in 38.3%, 37.5%, 34.8%, 33.7%, 31.4%, 28.7% VCs for MRI slice thicknesses of 1 mm, 2 mm, 3 mm, 4 mm, 5 mm and 6 mm respectively (Fig. 1) and in 19.3% of the VCs in radiography, *p* < 0.05 for all MRI slice thicknesses comparisons, with exception the comparison between MRI slices of 1–2 mm (*p* = 0.251) and 3–4 mm (*p* = 0.062). The anterior superior corners of Th12 and L1 were the overall most frequently affected site in both MRI and radiography (Supplementary Table 2). Upon consensus, radiography could detect 233 syndesmophytes, (Supplementary Table 2). MRI at any slice thickness could not detect 49/233 (21%) – 73/233 (31.3%) (all slice thicknesses) of the radiography syndesmophytes, while 186 (53.8%) – 279 (60.5%) of the syndesmophytes seen on MRI were not seen on radiography (Table 2). The anterior inferior corner of Th11, the posterior superior corner of L1 and the anterior inferior corner of L4 were the sites with the most syndesmophytes missed by MRI, as compared to radiography (data not shown). Overall, best agreement (either detected or missed in both MRI and radiography) between imaging modalities was found in the thicker slices (Fig. 1; Table 2). However, when assessing with radiography as a gold standard, more radiography detected syndesmophytes were missed on MRI slice thickness of 5 mm and 6 mm as compared to 1 mm slice thickness (missing syndesmophytes in MRI 64 vs. 51, *p* = 0. 035 and 73 vs. 51, *p* = 0.001, respectively). The same result was found for 4 mm, 5 mm and 6 mm slice thickness compared to 2 mm (missing syndesmophytes 58 vs. 49, *p* = 0. 039 and 64 vs. 49, *p* = 0.007, 73 vs. 49, *p* = 0.001, respectively). Additionally, with slice thickness of 3 mm less radiography detected syndesmophytes compared to 5 mm (52 vs. 64, *p* = 0.011) and with a 3 mm and 4 mm slice thickness less than with 6 mm (52 vs. 73, *p* = 0.005 and 58 vs. 73, *p* = 0.005, respectively) were missed (Table 2). In addition, more discrepant cases between MRI and radiography were found in MRI-detected syndesmophytes without concomitant FL (197–147, 59.7% − 56.8% of all MRI detected syndesmophytes) as compared with those with concomitant FL (133 − 112, 40.3% − 43.2% of all MRI detected syndesmophytes) for MRI slice thickness 1–6 mm, respectively.

**Table 2:** Agreement of radiography and MRI for syndesmophyte detection per MRI slice thickness

		Radiography		Agreement radiography/MRI	False positive MRI based on radiography as gold standard
**MRI**		yes	no		
**1 mm**	yes	182	279	72,6%	60.5%
	no	51	692		
**2 mm**	yes	184	267	73,8%	59.2%
	no	49	704		
**3 mm**	yes	181	238	75,9%	56.8%
	no	52	733		
**4 mm**	yes	175	231	76,0%	56.9%
	no	58	740		
**5 mm**	yes	169	209	77,3%	55.3%
	no	64	762		
**6 mm**	yes	160	186	78,5%	53.8%
	no	73	785		

MRI: Magnetic Resonance Imaging

### Exam and scoring duration of spine MRI for each slice thickness

Taking into account that thinner MRI slices could detect more syndesmophytes than the thicker ones and radiography, we assessed if performing a thinner slice MRI is also more time consuming. The time needed for this MRI protocol including every slice thickness was about 45 minutes (mins) (8–10 mins and about 4 mins for each one of the thinner and thicker slices, respectively).

The time for evaluation of the MR images for the presence of syndesmophytes and fat was 4’12’’, 4’11’’, 3’33’’, 2’27’’, 2’27’’ for 1 mm, 2 mm, 3 mm, 4 mm, 5 mm, and 6 mm slices, respectively, suggesting that a six times thinner slice does not increase the time to score fat and syndesmophytes by six times.

### The influence of fat lesions in the detection of syndesmophytes on MRI in comparison to radiography

**Fig. 2:**
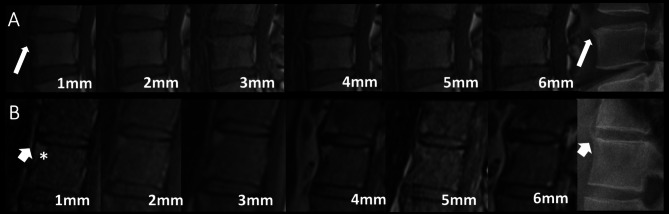
(**A**) Syndesmophyte at the anterior superior corner of L4 detected on both each MRI slice thickness and radiography (thin arrow). No concurrent presence of fat at the same corner. (**B**) Syndesmophyte at the anterior superior corner of Th12 detected on both each MRI slice thickness and radiography (thick arrow). Concurrent presence of fat at the same corner in MRI (asterisk) L: Lumbar; MRI: Magnetic Resonance Imaging; Th: Thoracic

Fat lesions were detected in 38.2%, 38.9%, 39.2%, 39.2%, 38.9%, 38.9% of vertebrae (lumbar or thoracic) with MRI slice thicknesses of 1–6 mm, respectively. MRI slice thickness had no relation to the frequency of detecting of fat lesions both in the thoracic and lumbar vertebrae. Fat lesions at the same VC with syndesmophytes were detected at 43.8%, 45.7%, 46.3%, 45.1%, 46.6% and 50.6% of the VCs affected with syndesmophytes for slice thicknesses of 1–6 mm respectively. The percentages of the VCs affected with syndesmophytes in MRI, with concurrent fat lesions presence, also having a syndesmophyte at the same vertebral corner in radiography were 39.6%, 41.7%, 43.3%, 43.7%, 44.9% and 44.6% for slice thicknesses of 1–6 mm respectively. When trying to evaluate if presence of fat in MRI at the same VC with a radiography detected syndesmophyte could facilitate the detection of this syndesmophyte in MRI, we found that the concurrent presence of fat and MRI syndesmophytes in the same vertebral corner and same MRI slice thickness with radiography detected syndesmophytes was found in 78.4%, 73.5%, 73.1%, 70.7%, 68.8% and 69.9% in slice thicknesses of 1–6 mm, respectively. Hence, about 66%-80% could be depicted in the presence of fat, suggesting that concurrent presence of fat lesions at the same corner could possibly facilitate the identification of a syndesmophyte at the same corner. Typical examples of syndesmophytes detected both on MRI and radiography at absence or presence of fat are shown in Fig. 2.

## Discussion

In this study, which aimed to analyze for the first time the sensitivity and specificity of spinal MR images for detection syndesmophytes using different thickness of slices, we report that MRI at any slice thickness detected more syndesmophytes than those detected in radiography. Overall, the thinner the slice thickness was, the more syndesmophytes were detected on MRI. However, in comparison to radiography as the ‘gold standard’ for detecting syndesmophytes, ‘false-positive’ results with MRI in our study seemed to increase parallel to decrease of slice thickness.

MRI cannot easily differentiate tissues with low proton density such as cortical bone and paravertebral ligaments since they have low or no signal intensity in almost all MRI sequences [13]. On the other hand one could think that radiography has lower sensitivity than MRI to detect such structural changes in their ‘earlier’ development phases and it’s rather the syndesmophytes detected in MRI that represent the ‘true picture’ of new bone formation. Performing a spine CT could provide answers regarding this hypothesis. Although not included as a first-line imaging method in any set of recommendations due to the high degree of radiation and higher costs, CT is a more precise method than radiography at detecting structural progress of the spine. Actually, the limitations of radiography regarding detection of radiographic progression within a short time of follow-up, led to the utilization of low-dose CT as a technique for detection of progression of bone formation. MRI- based synthetic CT (sCT) has also shown much higher specificity and sensitivity than radiography at detecting new bone formation in axSpA, with the overall mean number of scored lesions being close to those scored on low-dose CT [14]. Additionally, fat lesions in MRI have been positively corelated with CT detected syndesmophytes development [15].

In our cohort the anterior superior corner of L1 and Th12 were the most frequently affected sites with syndesmophytes in MRI. These data are in line with radiography [11] and CT [16] data that showed that the thoracolumbar area is the most frequently affected area in patients with r-axSpA and also the area that is most sensitive to depict radiographic progression over time [16].

Fat lesions in the bone marrow of patients with axSpA could represent tissue repair after inflammation, leading to development of new syndesmophytes [17]. Contrary to its role in syndesmophytes detection, MRI slice thickness had no influence on detecting fat lesions. Our data suggest that there is no optimal thickness for fat lesions detection. However, the presence of fat lesions was associated with a more accurate detection of syndesmophytes in comparison to radiography. This was not surprising, since the presence of the high signal intensity due to the fat lesion which is frequently extending into the newly formed syndesmophyte makes this bone structure much easier to depict.

Our findings suggest that although detecting more syndesmophytes than radiography, MRI still fails to detect up to one third of the syndesmophytes detected on the radiography as a gold-standard. Thinner MRI slices could detect numerically more syndesmophytes that were found to be present in MRI slice thicknesses of 3–4 mm, as suggested by international recommendations, seem to miss one fifth to one fourth of radiography-detected syndesmophytes [18].

In a real clinical setting, time is important for the evaluation of imaging examinations. Performing an MRI of thinner slices did not increase the time of evaluation of structural changes dramatically.

A limitation of the study is the absence of CT examinations for comparison to the used modalities. The higher sensitivity and specificity of CT vs. radiography for detection of syndesmophytes in patients with axSpA has been shown in previous studies [16]. It is therefore unknown, whether higher concordance or discordance with the MRI findings would have resulted if CT examinations of the same patients would have been performed. Nevertheless, since still radiography is established as the most commonly used examination for assessment of syndesmophytes in patients with axSpA, we believe that the data presented here are of value for the understanding of such spinal structures in daily practice and when both radiography but also MRI examinations are evaluated.

In conclusion, our data suggest that MRI slice thickness plays a role in detecting syndesmophytes, with thinner slice thicknesses detecting more syndesmophytes compared to the thicker ones, although overall MRI may still be considered as depicting rather unclear findings due to the missing correlation to radiography as the gold standard. Slice thickness does not seem to affect the detection of fat lesions, while caution should be paid in syndesmophytes that are depicted in the absence of fat lesions due to the uncertainty of these syndesmophytes in comparison to the more accurately detected ones when fat is present. Prospective studies in combination with CT to confirm these results are warranted to make any recommendation for daily practice.

## Supplementary Information

Below is the link to the electronic supplementary material.

Supplementary Material 1

Supplementary Material 2

## Data Availability

Data is provided within the manuscript or supplementary information files.

## Abbreviations

BASFI: Bath Ankylosing Spondylitis Functional Index
BME: Bone marrow edema
CT: Computed tomography
FL: Fat lesions
IQR: Interquartile range L: lumbar
MRI: Magnetic resonance imaging
mSASSS: Modified Stoke Ankylosing Spondylitis Spine Score
nr-axSpA: Non-radiographic axial Spondyloarthritis
r-axSpA: Radiographic axial Spondyloarthritis
SD: Standard deviation
SIJs: Sacroiliac joints
SNR: Signal-to-Noise Ratio
Th: Thoracic
TSE: Turbo spin echo
T1W: T1-weighted
VC: Vertebral corner
