# Supplementary material for: The impact of MRI slice thickness on the detection of spinal syndesmophytes in axial spondyloarthritis
Source: Arthritis Res Ther. 2025 Nov 14;27:212. doi: 10.1186/s13075-025-03665-x (PMC12619499; doi:10.1186/s13075-025-03665-x)
Supplement: Supplementary file 1 — Supplementary Material 1 [file 13075_2025_3665_MOESM1_ESM.docx]

Supplementary Table 1. Magnetic Resonance Imaging (MRI) Sequence Parameters

| Sequence | T1W TSE | T2W TSE |
| --- | --- | --- |
| Imaging plane | sagittal | sagittal |
| TE/TR (ms) | 12/771 | 95/3500 |
| Refocusing Flip Angle (°) | 120 | 160 |
| Slice thickness (mm) | 1 | 4 |
| FoV (mm x mm) | 300 x 300 | 259 x 259 |
| Acquisition Matrix | (320/0/0/224) | (384/0/0/288) |
| Spacing between slices (mm) | 1 | 4.8 |
| Acquisition time (min:sec) | 9:43 | 3:48 |
| FoV: Field of View; T1W: T1-weighted; T2W: T2-weighted;TE: Echo time; TR: Repetition time; TSE: Turbo Spin Echo | | |
