# Supplementary material for: The impact of MRI slice thickness on the detection of spinal syndesmophytes in axial spondyloarthritis
Source: Arthritis Res Ther. 2025 Nov 14;27:212. doi: 10.1186/s13075-025-03665-x (PMC12619499; doi:10.1186/s13075-025-03665-x)
Supplement: Supplementary file 2 — Supplementary Material 2 [file 13075_2025_3665_MOESM2_ESM.docx]

| Supplementary Table 2. Distribution of syndesmophytes in different MRI slice thicknesses and radiography | |
| --- | --- |
| scored corner/slice thickness | syndesmophytes n,(%) |
| Th11_as_1mm  Th11_ai_1mm  Th11_ps_1mm  Th11_pi_1mm | 13 (30.2)  28 (65.1)  9 (20.9)  17 (39.5 |
| Th11_as_2mm  Th11_ai_2mm  Th11_ps_2mm  Th11_pi_2mm | 14 (32.6)  25 (58.1)  6 (64.1)  19 (44.2) |
| Th11_as_3mm  Th11_ai_3mm  Th11_ps_3mm  Th11_pi_3mm | 12 (27.9)  23 (53.5)  6 (14)  18 (41.9) |
| Th11_as_4mm  Th11_ai_4mm  Th11_ps_4mm  Th11_pi_4mm | 12 (27.9)  24 (55.8)  5 (11.6)  14 (32.6) |
| Th11_as_5mm  Th11_ai_5mm  Th11_ps_5mm  Th11_pi_5mm | 11 (25.6)  22 (51.2)  5 (11.6)  12 (27.9) |
| Th11_as_6mm  Th11_ai_6mm  Th11_ps_6mm  Th11_pi_6mm | 8 (18.6)  21 (48.8)  5 (11.6)  12 (27.9) |
| Th11_as_radiography  Th11_ai_radiography  Th11_ps_radiography  Th11_pi_radiography | 5 (11.6)  12 (27.9)  4 (9.3)  3 (7) |
| Th12_as_1mm  Th12_ai_1mm  Th12_ps_1mm  Th12_pi_1mm | 38 (88.4)  28 (65.1)  13 (30.2)  18 (41.9) |
| Th12_as_2mm  Th12_ai_2mm  Th12_ps_2mm  Th12_pi_2mm | 31 (72.1)  24 (55.8)  13 (30.2)  19 (44.2) |
| Th12_as_3mm  Th12_ai_3mm  Th12_ps_3mm  Th12_pi_3mm | 28 (65.1)  24 (55.8)  14 (32.6)  19 (44.2) |
| Th12_as_4mm  Th12_ai_4mm  Th12_ps_4mm  Th12_pi_4mm | 28 (65.1)  24 (55.8)  12 (27.9)  18 (41.9) |
| Th12_as_5mm  Th12_ai_5mm  Th12_ps_5mm  Th12_pi_5m | 28 (65.1)  22 (51.2)  10 (23.3)  14 (32.6) |
| Th12_as_6mm  Th12_ai_6mm  Th12_ps_6mm  Th12_pi_6mm | 27 (62.8)  21 (48.8)  11 (25.6)  13 (30.2) |
| Th12_as_radiography  Th12_ai_ radiography  Th12_ps_radiography  Th12_pi_radiography | 17 (39.5)  16 (37.2)  7 (16.3)  3 (7) |
| L1_as_1mm  L1_ai_1mm  L1_ps_1mm  L1_pi_1mm | 36 (83.7)  22 (51.2)  9 (20.9)  20 (46.5) |
| L1_as_2mm  L1_ai_2mm  L1_ps_2mm  L1_pi_2mm | 35 (81.4)  18 (41.9)  11 (25.6)  19 (44.2) |
| L1_as_3mm  L1_ai_3mm  L1_ps_3mm  L1_pi_3mm | 34 (79.1)  18 (41.9)  12 (27.9)  17 (39.5) |
| L1_as_4mm  L1_ai_4mm  L1_ps_4mm  L1_pi_4mm | 32 (74.4)  19 (44.2)  8 (18.6)  20 (46.5) |
| L1_as_5mm  L1_ai_5mm  L1_ps_5mm  L1_pi_5mm | 32 (74.4)  20 (46.5)  7 (16.3)  17 (39.5) |
| L1_as_6mm  L1_ai_6mm  L1_ps_6mm  L1_pi_6mm | 28 (65.1)  18 (41.9)  6 (14)  16 (37.2) |
| L1_as_radiography  L1_ai_radiography  L1_ps_radiography  L1_pi_radiography | 18 (41.9)  11 (25.6)  8 (18.6)  5 (11.6) |
| L2_as_1mm  L2_ai_1mm  L2_ps_1mm  L2_pi_1mm | 28 (65.1)  20 (46.5)  11 (25.6)  14 (32.6) |
| L2_as_2mm  L2_ai_2mm  L2_ps_2mm  L2_pi_2mm | 28 (65.1)  17 (39.5)  11 (25.6)  13 (30.2) |
| L2_as_3mm  L2_ai_3mm  L2_ps_3mm  L2_pi_3mm | 26 (60.5)  15 (34.9)  7 (16.3)  9 (20.9) |
| L2_as_4mm  L2_ai_4mm  L2_ps_4mm  L2_pi_4mm | 26 (60.5)  16 (34.9)  7 (16.3)  10 (20.9) |
| L2_as_5mm  L2_ai_5mm  L2_ps_5mm  L2_pi_5mm | 26 (60.5)  13 (30.2)  5 (11.6)  11(25.6) |
| L2_as_6mm  L2_ai_6mm  L2_ps_6mm  L2_pi_6mm | 22 (51.2)  13 (30.2)  3 (7)  9 (20.9) |
| L2_as_radiography  L2_ai_radiography  L2_ps_radiography  L2_pi_radiography | 13 (30.2)  14 (32.6)  6 (14)  2 (4.7) |
| L3_as_1mm  L3_ai_1mm  L3_ps_1mm  L3_pi_1mm | 20 (46.5)  13 (30.2)  8 (18.6)  10 (23.3) |
| L3_as_2mm  L3_ai_2mm  L3_ps_2mm  L3_pi_2mm | 23 (53.5)  13 (30.2)  9 (20.9)  11 (25.6) |
| L3_as_3mm  L3_ai_3mm  L3_ps_3mm  L3_pi_3mm | 23 (53.5)  13 (30.2)  8 (18.6)  9 (20.9) |
| L3_as_4mm  L3_ai_4mm  L3_ps_4mm  L3_pi_4mm | 22 (51.2)  13 (30.2)  7 (16.3)  8 (18.6) |
| L3_as_5mm  L3_ai_5mm  L3_ps_5mm  L3_pi_5mm | 21 (48.8)  12 (27.9)  6 (14)  9 (20.9) |
| L3_as_6mm  L3_ai_6mm  L3_ps_6mm  L3_pi_6mm | 20 (46.5)  12 (27.2)  5 (11.6)  8 (18.6) |
| L3_as_radiography  L3_ai_radiography  L3_ps_radiography  L3_pi_radiography | 16 (37.2)  8 (18.6)  3 (7)  5 (11.2) |
| L4_as_1mm  L4_ai_1mm  L4_ps_1mm  L4_pi_1mm | 23 (53.2)  8 (18.6)  6 (14)  10 (23.3) |
| L4_as_2mm  L4_ai_2mm  L4_ps_2mm  L4_pi_2mm | 25 (58.1)  10 (23.3)  10 (23.3)  8 (18.6) |
| L4_as_3mm  L4_ai_3mm  L4_ps_3mm  L4_pi_3mm | 25 (58.1)  10 (23.3)  9 (20.9)  8 (18.6) |
| L4_as_4mm  L4_ai_4mm  L4_ps_4mm  L4_pi_4mm | 23 (53.5)  9 (20.9)  7 (16.3)  9 (20.9) |
| L4_as_5mm  L4_ai_5mm  L4_ps_5mm  L4_pi_5mm | 21 (48.8)  8 (18.6)  7 (16.3)  8 (18.6) |
| L4_as_6mm  L4_ai_6mm  L4_ps_6mm  L4_pi_6mm | 18 (41.9)  7 (16.3)  6 (14)  7 (16.3) |
| L4_as_radiography  L4_ai_radiography  L4_ps_radiography  L4_pi_radiography | 17 (39.5)  11 (25.6)  5 (11.6)  3 (7) |
| L5_as_1mm  L5_ai_1mm  L5_ps_1mm  L5_pi_1mm | 16 (37.2)  9 (20.9)  9 (20.9)  5 (11.6) |
| L5_as_2mm  L5_ai_2mm  L5_ps_2mm  L5_pi_2mm | 16 (37.2)  9 (20.9)  9 (20.9)  5 (11.6) |
| L5_as_3mm  L5_ai_3mm  L5_ps_3mm  L5_pi_3mm | 13 (30.2)  8 (18.6)  7 (16.3)  4 (9.3) |
| L5_as_4mm  L5_ai_4mm  L5_ps_4mm  L5_pi_4mm | 13 (30.2)  8 (18.6)  8 (18.6)  4 (9.3) |
| L5_as_5mm  L5_ai_5mm  L5_ps_5mm  L5_pi_5mm | 13 (30.2)  8 (18.6)  7 (16.3)  3 (7) |
| L5_as_6mm  L5_ai_6mm  L5_ps_6mm  L5_pi_6mm | 13 (30.2)  9 (20.9)  6 (14)  2 (4.7) |
| L5_as_radiography  L5_ai_radiography  L5_ps_radiography  L5_pi_radiography | 10 (23.3)  7 (16.3)  3 (7)  1 (2.3) |
| ai, anterior inferior; as, anterior superior; L, lumbar; MRI, magnetic resonance imaging; ps, posterior superior; pi, posterior inferior; Th, thoracic | |
